# Breast Cancer MCF-7 Cell Spheroid Culture for Drug Discovery and Development

**DOI:** 10.4236/jct.2022.133009

**Published:** 2022-03-09

**Authors:** Guangping Chen, William Liu, Bingfang Yan

**Affiliations:** 1 Department of Physiological Sciences, College of Veterinary Medicine, Oklahoma State University, Stillwater, OK, USA; 2 College of Pharmacy, University of Cincinnati, Cincinnati, OH, USA

**Keywords:** MCF-7 Cell, Spheroid Culture, 3D Cell Culture, Estrogen-Dependent Breast Cancer, Cancer Drug Development, Personalized Cancer Drug Development

## Abstract

*In vitro* 3D cancer spheroids (tumoroids) exhibit a drug resistance profile similar to that found in solid tumors. 3D spheroid culture methods recreate more physiologically relevant microenvironments for cells. Therefore, these models are more appropriate for cancer drug screening. We have recently developed a protocol for MCF-7 cell spheroid culture, and used this method to test the effects of different types of drugs on this estrogen-dependent breast cancer cell spheroid. Our results demonstrated that MCF-7 cells can grow spheroid in medium using a low attachment plate. We managed to grow one spheroid in each well, and the spheroid can grow over a month, the size of the spheroid can grow over a hundred times in volume. Our targeted drug experimental results suggest that estrogen sulfotransferase, steroid sulfatase, and G protein-coupled estrogen receptor may play critical roles in MCF-7 cell spheroid growth, while estrogen receptors *α* and *β* may not play an essential role in MCF-7 spheroid growth. Organoids are the miniatures of *in vivo* tissues and reiterate the *in vivo* microenvironment of a specific organ, best fit for the *in vitro* studies of diseases and drug development. Tumoroid, developed from cancer cell lines or patients’ tumor tissue, is the best *in vitro* model of *in vivo* tumors. 3D spheroid technology will be the best future method for drug development of cancers and other diseases. Our reported method can be developed clinically to develop personalized drugs when the patient’s tumor tissues are used to develop a spheroid culture for drug screening.

## 1. Introduction

About 1 in 8 women will develop invasive breast cancer at some point in their lives [1] [2]. It is the most common cause of cancer death among women worldwide (over 520,000 death each year) [3] [4], including more than 40,000 deaths each year in the US alone [5]. Despite advances in treatment and improvement in survival over the past several decades, additional research is needed to further reduce the personal and societal toll of this disease.

The majority of breast cancers are estrogen-dependent [6] [7] [8], occurring when estrogen-sensitive tissues proliferate in response to estrogens and undergo tumorigenesis [9]. Several categories of drugs have been developed to treat these cancers, including estrogen-blocking drugs such as estrogen receptors (ERs) blockers [10] [11] and inhibitors of estrogen synthesis enzymes such as aromatase and steroid sulfatase (STS) [12] [13] [14] [15], with mixed results. Despite its role in regulating estrogenic activity, estrogen sulfotransferase (SULT1E1) has not yet been fully investigated as a therapeutic target in estrogen-dependent cancers [16].

Cell culture is a widely used *in vitro* tool for biomedical research, drug discovery, and drug development. For decades, researchers have used 2D cell culture in which cells grow as monolayers on plate surfaces [17]. 3D cell culture has become more and more a focus of research in cancer drug discovery and development recently. *In vitro* 3D cancer cell spheroids (tumoroids) exhibit a drug resistance profile similar to that found in solid tumors. Patient-derived (or animal tissue-derived) organoids (derived from stem cells or other progenitor cells) also more closely mimic *in vivo* conditions for personalized drug development. 3D spheroid culture methods recreate more physiologically relevant microenvironments for cells. Therefore, these models are more appropriate for cancer and other disease drug screening. Methods for creating 3D cell cultures are usually complex and expensive, including commercially available matrix materials such as hydrogels and peptide hydrogels. In contrast, recently developed round-bottom low-attachment plate methods provide a more convenient, less expensive approach. MCF-7 is a commonly used breast cancer cell line for breast cancer research for more than 40 years by multiple research groups, it is an estrogen-dependent breast cancer cell line [18]. Recently, MCF-7 cells have been used to develop tumoroids in ultra-low attachment 6-well plates in culture medium [19], resulting in new insights about spatio-temporal arrangements of tunneling nanotubes, amyloid fibrils, cell connections, and cellular bridges. We have recently developed a simple, inexpensive method for MCF-7 cell spheroid growth in 96-well low attachment plates. We used this method to test breast cancer drugs for their effect on the MCF-7 spheroid. Our results demonstrate that MCF-7 cell can grow in a plate like a solid tumor, and this method can be used for cancer drug testing and mechanistic studies. Spheroid culture method could be developed to screen personalized drugs for individual patients clinically.

## 2. Materials and Methods

### 2.1. Materials

Phenol red free cell culture medium Dulbecco’s Modified Eagle Medium (DMEM) was from Life Technologies Corporation (Carlsbad, CA 92008, USA) (Catalog Number 31053036, lot number 2193168). Fetal bovine serum was purchased from Quality Biological Inc. 100× Penicillin/Streptomycin solution, 100× glutamine solution, and 100 X trypsin/EDTA solution were ordered from Thermo-Fisher Scientific with the highest quality available. Estradiol, tamoxifen, and quercetin were order from Sigma-Aldrich. STX64 (STX, Irosustat, Cat. No BD767626) was ordered from BLD Pharmatech Co., Limited. G-1 (Cat. No. HY-107216), G15 (Cat. No. HY-103449), Propyl pyrazole triol (PPT, Cat. No. HY-100689) and Diarylpropionitrile (DPN, Cat. No. HY-12452) were purchased from Med-ChemExpress (Monmouth Junction, NJ 08852, USA). All other chemicals were purchased with highest grade. U-bottom, clear, cellstar^®^, cell-repellent surface 96-well plates were purchased from Greiner Bio-One (Frickenhausen, Germany).

### 2.2. MCF-7 2D Cell Culture

Human MCF-7 cells were obtained from American Type Culture Collection (ATCC, HTB-22). Cells were initially seeded in 25 cm^2^ culture flasks (Corning), and sub-cultured at a ratio of 1:3 once a confluence of 80% – 90% was reached. Culture procedures provided by the company were used. Phenol red free DMEM medium with 10% FBS, 0.01 mg/ml bovine insulin, 10 nM estradiol, and standard penicillin/streptomycin and glutamine was used for both 2D and spheroid culture of MCF-7 cells.

### 2.3. MCF-7 Cell Spheroid Culture and Drug Treatment

U-bottom, clear, cell-repellent surface 96-well plates were used for spheroid culture of MCF-7 cells. MCF-7 cells were expanded using 2D cell culture. 500 to 5000 cells (in 200 ml of medium) were used to seed each well of the 96-well plates. The plates were centrifuged at 1000 RPM for 5 minutes after each time of operation before incubation in incubator. Three fourths (or one half) of the medium was very carefully removed every two days, and new medium was added. The plates were kept very still (no disturbance) before removing of medium, and the medium was removed carefully and slowly using a 200 ml multichannel pipette with an angle about 90 degree to the well, top of the tips in the middle of the medium. For drug treatment, all drug stock solutions were made in ethanol with 100× of the needed concentrations, drugs were added after each time of change of fresh medium.

### 2.4. Data Analysis

**Figure 1.**
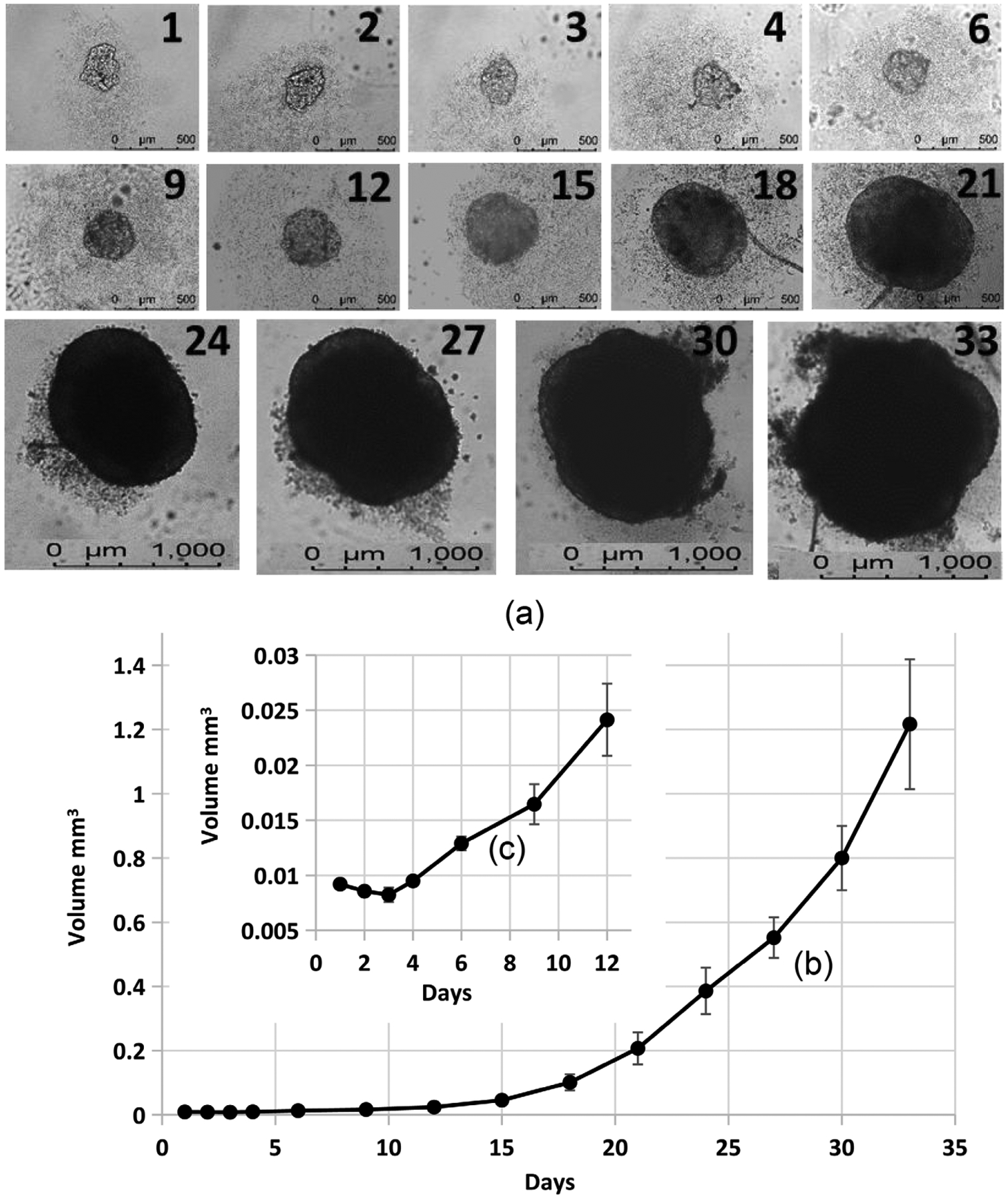
MCF-7 cell spheroid growth in a 96 well round bottom ultra-low attachment plate. 2000 cells/well were seeded. 100× microscope magnification was used between days 1 and 21. After 24 days, 40× microscope magnification was used. The pictures shown in (a) (an example of one spheroid) were adjusted to the same scale. Spheroid volume ((b) and (c)) was calculated using the ImageJ software. T-Test calculation results suggest that days 2, 3, and 4 are not significantly different from day 1 (p > 0.05), and all days above 6 days are significantly different from day 1 (p < 0.0001).

All experiments were done in sextuplicate and repeated three times. The volume of the spheroids was calculated using the free software ImageJ based on the scale in the pictures given by the microscope. Microsoft Excel was used for the statistical average and standard error calculations and figure plotting. T-Test method was used to calculate the statistical significance of difference between day 1 and other days in Figure 1. Two-way ANOVA method was used to calculate the statistical significance of difference between control group (ethanol treatment) and each drug concentrations treatment groups.

## 3. Results

### 3.1. MCF-7 Cell Spheroid Culture

We have developed a new protocol for growing MCF-7 cell spheroids using extra low attachment 96 well plates. MCF-7 cells were expanded using regular 2D cell culture. Five hundred to five thousand cells per well were used to seed extra low attachment 96 well plates. With one spheroid per well, we can maintain the spheroid culture for over 30 days. During the first three days, the cell aggregates do not grow (Figure 1(c)), then cell spheroids begin continue growing until they are terminated (Figure 1(b)). Figure 1(a) shows an example of a spheroid growth (2000 cells/well were seeded). 100× microscope magnification was used between days 1 and 21 to take pictures. After 24 days, 40× microscope magnification was used. The pictures shown in Figure 1(a) were adjusted to the same scale. Our results clearly indicate that MCF-7 cells can grow like a tumor *in vitro* in a low attachment plate in medium without attachment. Detailed structure of the spheroids (especially seen on the microscope computer screen) suggest that the spheroids are mostly free to move in the medium. This agrees with a publication [19]. Our protocol for producing and maintaining tumoroids can be used for cancer drug screening, discovery, and development. It can also be used for mechanistic studies for various types of cancers. Our results (Figure 1) and other recently published results [19] suggest MCF-7 spheroid development (formation) is mostly completed within 3 – 4 days. After 4 days, spheroids grow in medium similar to tumor growth.

### 3.2. Effect of Breast Cancer Drugs on MCF-7 Cell Spheroids

**Figure 2.**
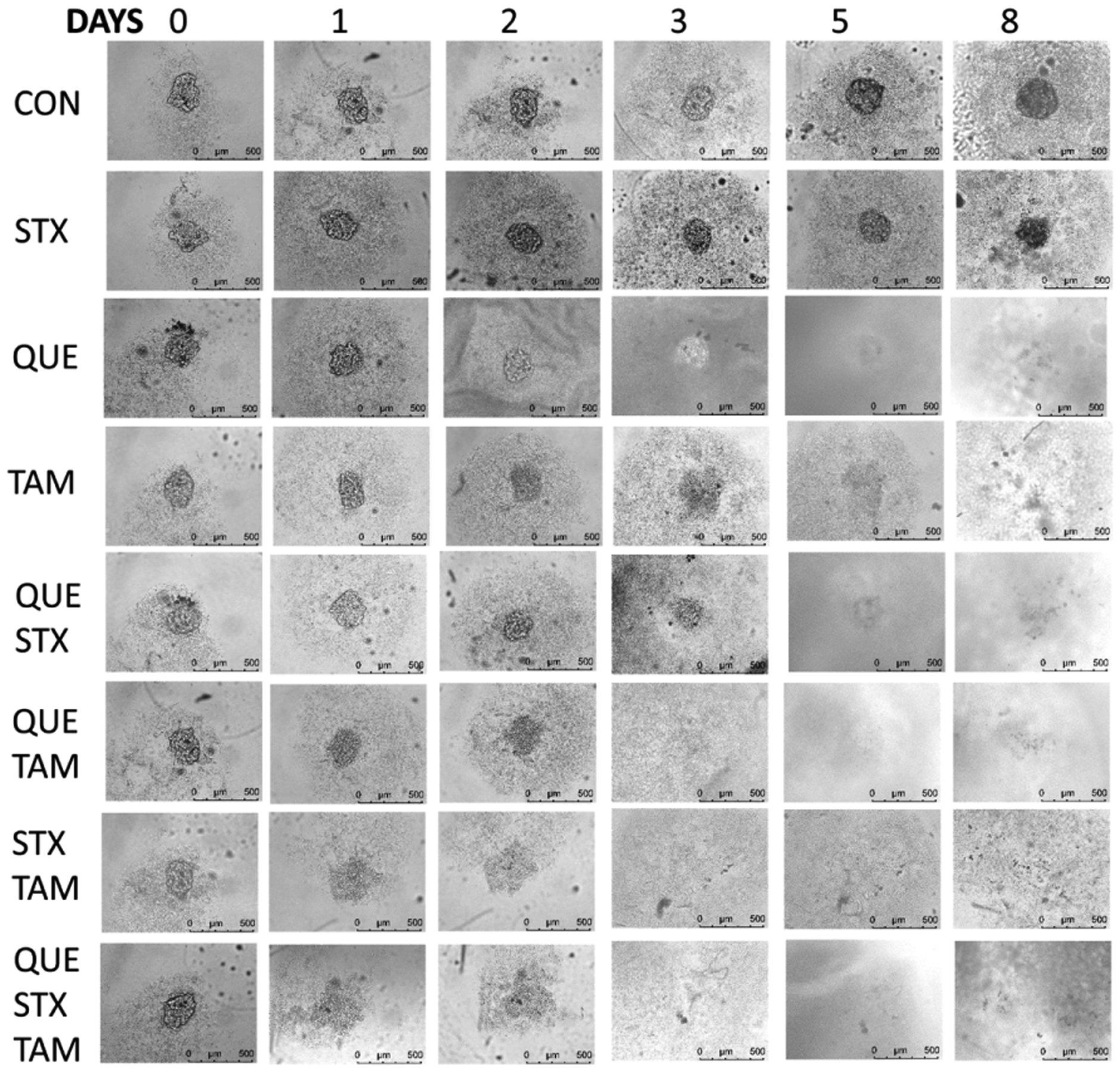
Effect of breast cancer drugs on MCF-7 Spheroid. 10 *μ*M of drugs was used for the treatment. MCF-7 spheroid was treated after 1 day of spheroid development. CON = ethanol control, STX = STX64 (Irosustat), QUE = quercetin, TAM = tamoxifen.

**Figure 3.**
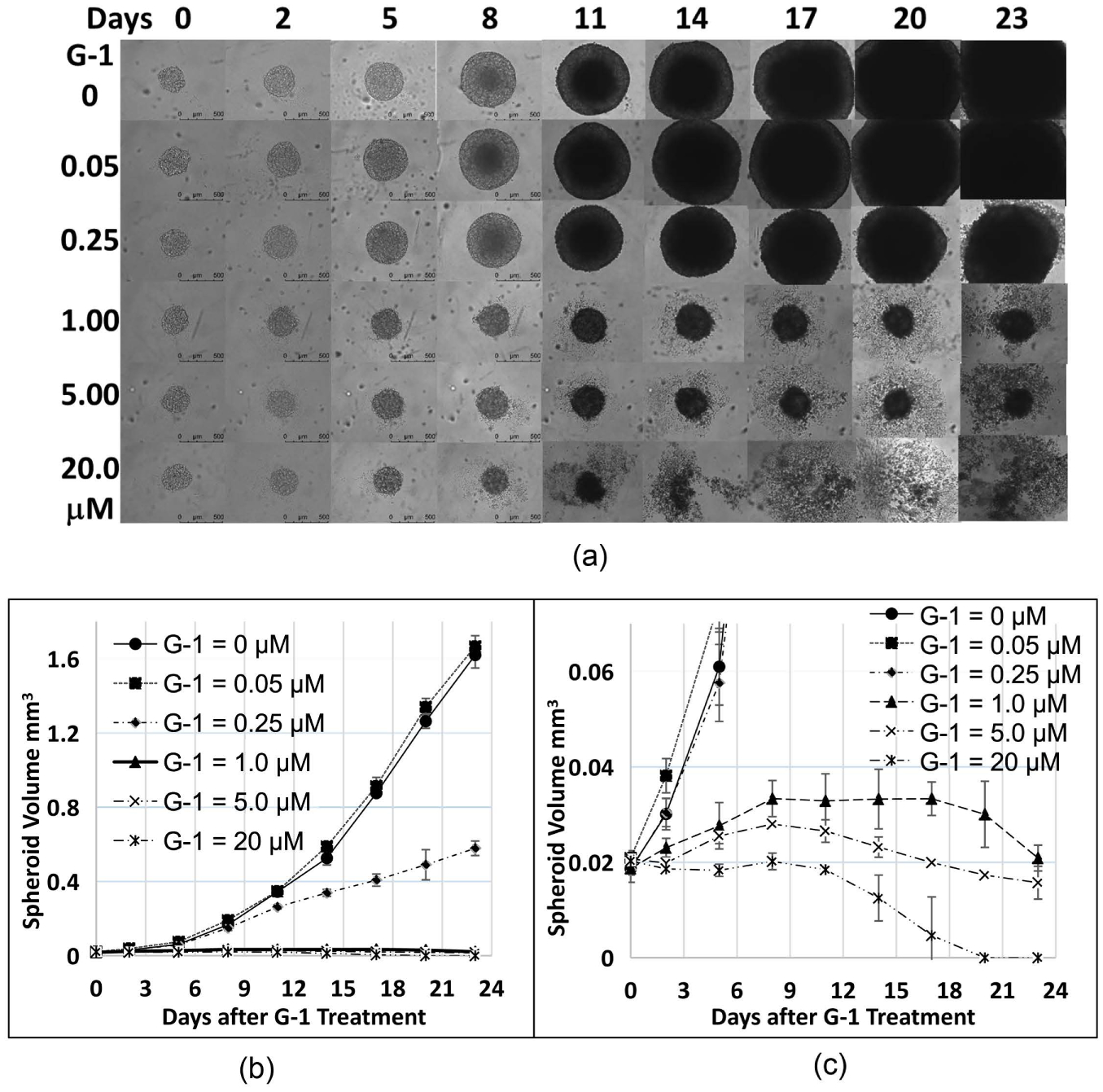
Time- and concentration-dependent Effect of G-1 on MCF-7 spheroid growth. MCF-7 spheroids were treated after 2 days of spheroid development. Two-way ANOVA calculation results suggest that G-1 = 0.05 *μ*M is not significantly different from the control (G-1 = 0) (p > 0.05). All other G-1 concentrations are significantly different from the control (p < 0.0001).

**Figure 4.**
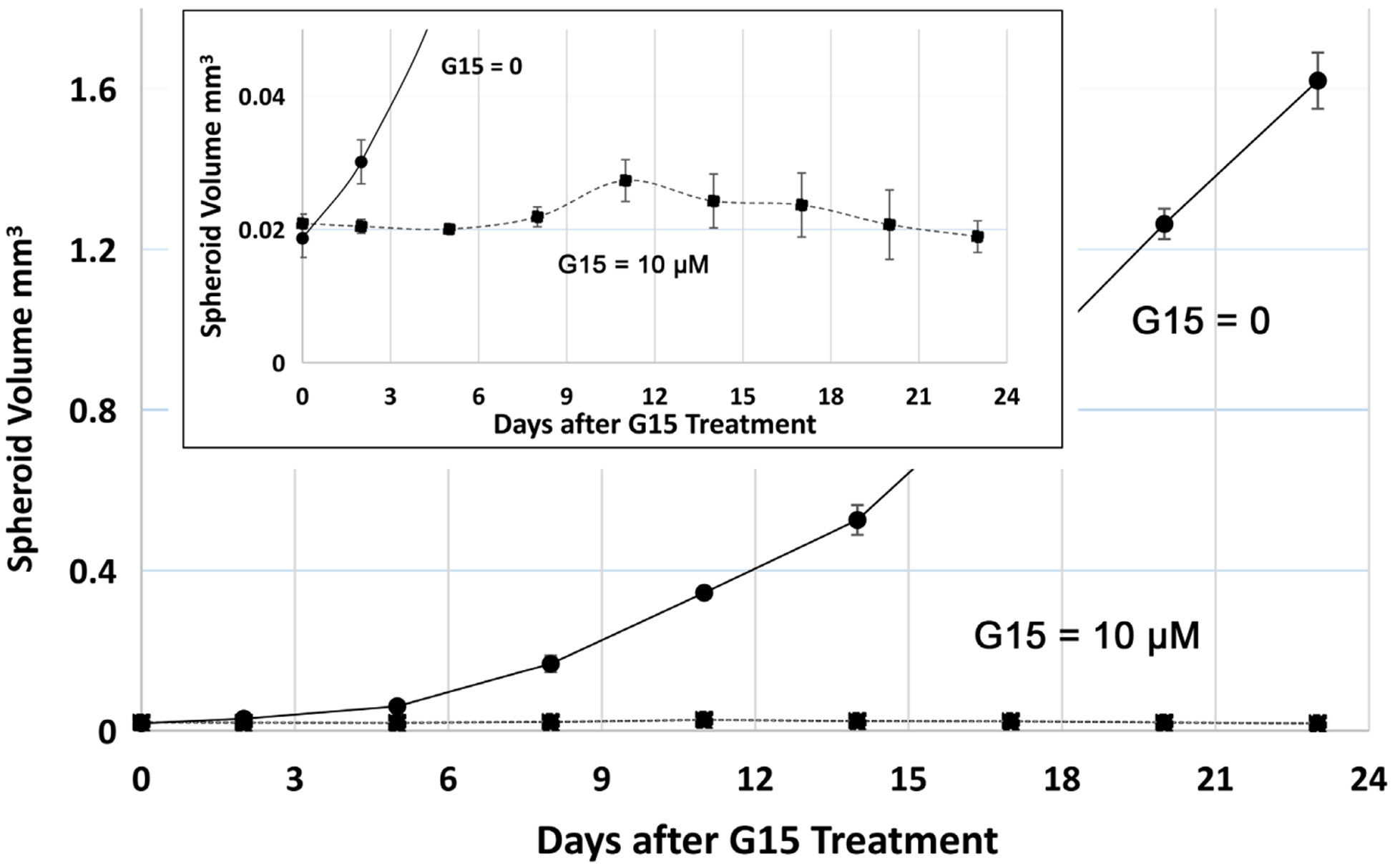
Effect of G15 on MCF-7 spheroid growth. 10 *μ*M of G15 was used to treat MCF-7 spheroid after 2 days of spheroid development. Two-way ANOVA calculation result suggests that G15 = 10 *μ*M is significantly different from the control (p < 0.0001).

**Figure 5.**
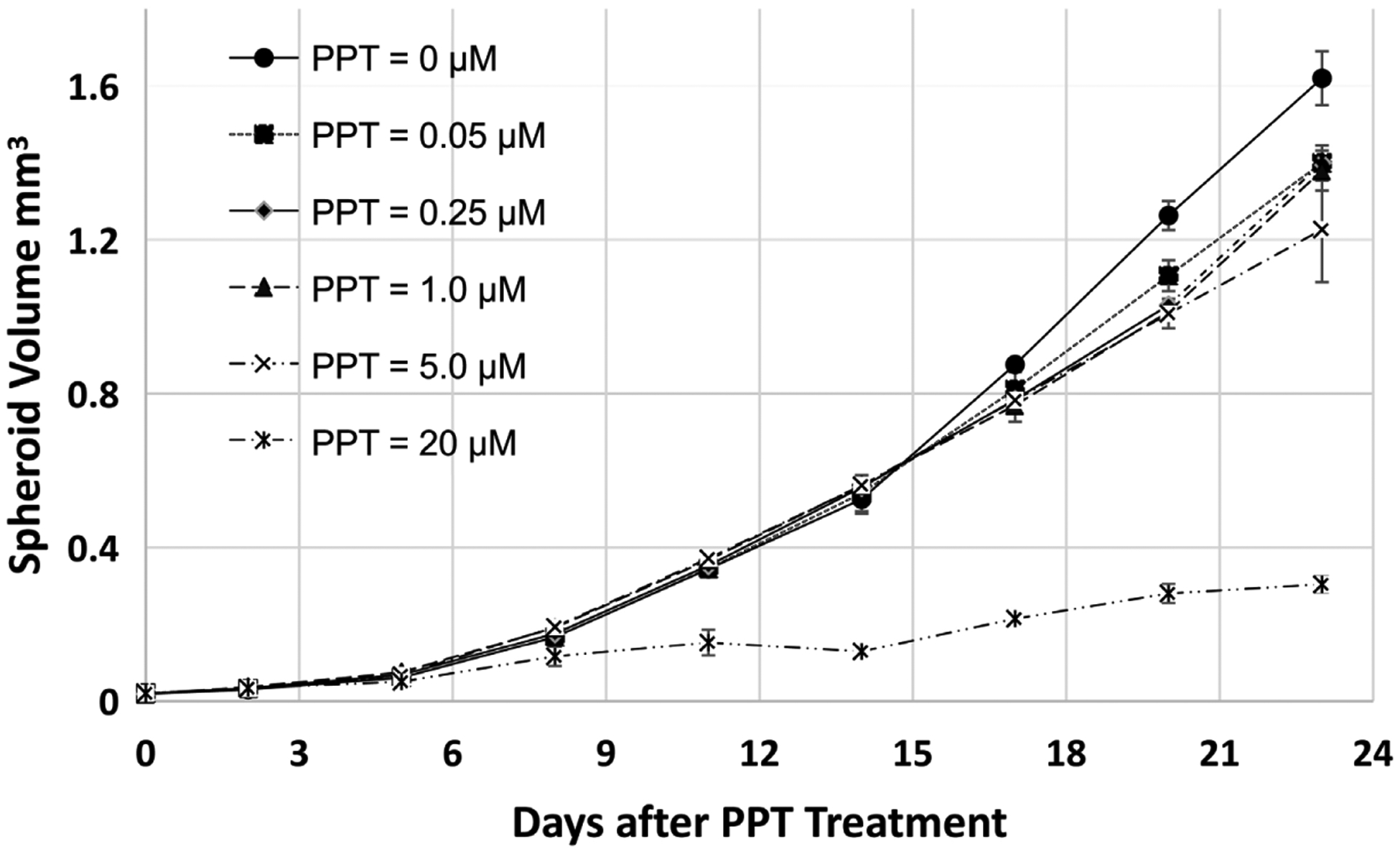
Time- and concentration-dependent Effect of PPT on MCF-7 spheroid growth. MCF-7 spheroids were treated after 2 days of spheroid development. Two-way ANOVA calculation results suggest that PPT treatments are significantly different from the control (p numbers between 0.05 and 0.0001) between concentrations 0.05 to 5.0 *μ*M. PPT = 20 *μ*M is significantly different from the control (p < 0.0001).

**Figure 6.**
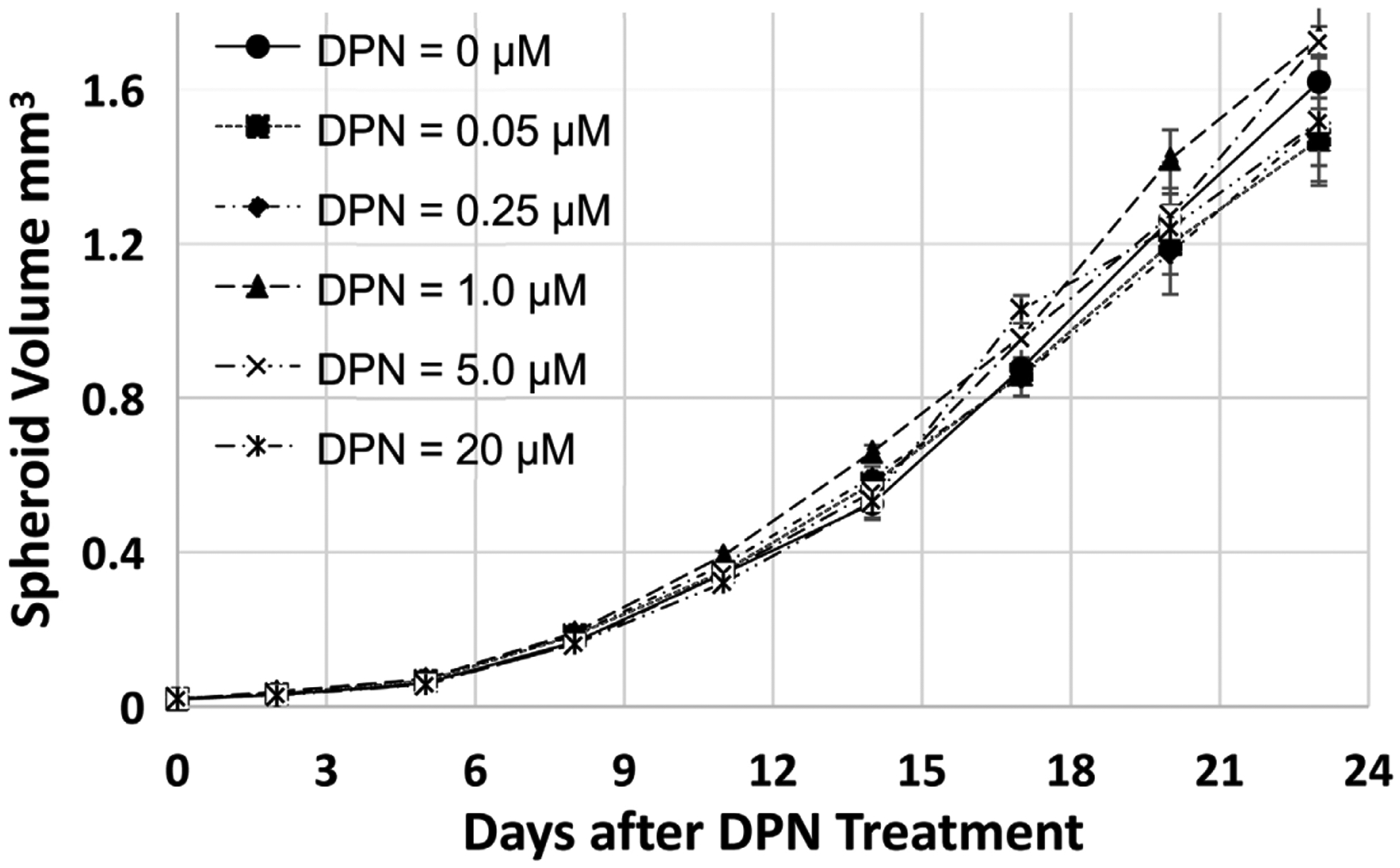
Time- and concentration-dependent Effect of DPN on MCF-7 Spheroid Growth. MCF-7 spheroids were treated after 2 days of spheroid development. Two-way ANOVA calculation results suggest that DPN treatments (all concentrations) are not significantly different from the control (p > 0.05).

We investigated the effect of breast cancer drugs on MCF-7 cell spheroids to validate the method. 2000 cells were seeded in each well of a 96 well round bottom extra low attachment plate (Figure 2). After 1 day, spheroids were treated with 10 *μ*M STX64 (STX), quercetin (QUE), tamoxifen (TAM) or different combinations (in sextuplicate). STS, an estrogen synthase, activates inactive estrogen sulfates (circulating form of estrogens) into active estrogens at targeting sites (estrogen is mostly synthesized in ovary), and its action is inhibited by STX. As shown in Figure 2, STX (10 *μ*M) inhibited MCF-7 spheroid growth compared to control (control plot similar as Figure 1(c), STX plot not shown), but it did not kill MCF-7 spheroids within a week. Our published results have shown that QUE up-regulates SULT1E1 expression and inhibits proliferation of MCF-7 cells in 2D culture [21]. As shown in Figure 2, QUE (10 *μ*M) completely killed MCF-7 spheroids within a week. QUE is a flavonoid, and its actions on MCF-7 cells may be more complex than just induce SULT1E1. Tamoxifen (TAM), an antagonist of ERs, was the very first breast cancer drug and is still the most commonly used breast cancer drug clinically. As shown in Figure 2, TAM (10 *μ*M) completely killed MCF-7 spheroids within a week. Our results suggest that STX64 (STS inhibitor) and TAM (antagonist of ERs) may have different mechanisms of cytotoxicity. STX64 decreases the active form of estrogens and thereby inhibits spheroid growth but does not cause spheroid death, while TAM causes spheroid (tumor) break down and cell death. The combination of different drugs had additive effects on the spheroid toxicity (Figure 2). Results shown in Figures 2–6 suggest that when spheroids were drug treated after 2 days of spheroid development (formation), the cytotoxicity of drugs to the spheroid is lower than when drug treatment started 1 day after spheroid development (Figure 1). We are currently investigating the effect of spheroid development time on drug effectiveness.

### 3.3. Time- and Concentration-Dependent Effect of G-1 on MCF-7 Spheroid Growth

When MCF-7 spheroids were treated with different concentrations of G-1, the specific antagonist of G protein-coupled estrogen receptor (GPER) [20], after two days of spheroid development, G-1 inhibited MCF-7 spheroid growth in a time- and concentration-dependent manner (Figure 3). Figure 3(a) shows one spheroid example for each concentration of G-1 used. To show most spheroids clearly, some of the bigger spheroids were not shown in all in the picture in Figure 3(a). Spheroid volumes (Figure 3(a) and Figure 3(b)) were calculated using the software of ImageJ. Three times of experimental results were used to calculate the average volume and standard error.

### 3.4. Effect of 10 *μ*M G15 on MCF-7 Cell Spheroid Growth

Our experiments results suggest that the antagonist of GPER, G15 [20], tremendously inhibited the MCF-7 spheroid growth (spheroid was treated with 10 *μ*M of G15 after 2 days of spheroid development) (Figure 4). This result agree with G-1 inhibition result, suggesting that GPER is critical for MCF-7 spheroid growth, Our results also show that the clinical breast cancer drug TAM significantly inhibited MCF-7 spheroid growth at the same condition as described above (Figure not shown). However, G-1 and G15 are much more effective than TAM at the same conditions. These experimental results further demonstrated that our spheroid culture method is a very promising method for cancer drug discovery and development. Cultured spheroid closely mimic *in vivo* solid tumor.

### 3.5. Time- and Concentration-Dependent Effect of PPT on MCF-7 Spheroid Growth

Results shown in Figure 5 suggest that PPT (propylpyrazoletriol), a specific estrogen receptor *α* (ER*α*) antagonist [21], did not significantly inhibit MCF-7 spheroid growth between concentration 0.05 to 5.0 *μ*M within two weeks, while it moderately (significant based on t-Test results) inhibited the spheroid growth after two weeks at these concentrations. PPT significantly inhibited MCF-7 spheroid growth at 20 *μ*M. The inhibition at high concentrations and or longer time treatments could be caused by its non-specific effect on other estrogen receptors at high concentrations. This result suggests that ERα may not play critical roles in MCF-7 spheroid growth.

### 3.6. Time- and Concentration-Dependent Effect of DPN on MCF-7 Spheroid Growth

Results shown in Figure 6 suggest that DPN (diarylpropionitrile), a specific estrogen receptor *β* (ER*β*) antagonist [22], did not significantly inhibit MCF-7 spheroid growth between concentration 0.05 to 20 *μ*M. This result suggests that ER*β* may not play critical roles in MCF-7 spheroid growth.

## 4. Discussion

Estrogen-dependent breast cancer MCF-7 cells have been used to develop spheroid (tumoroids) in ultra-low attachment 6-well plates in culture medium, resulting in new insights about spatio-temporal arrangements of tunneling nanotubes, amyloid fibrils, cell connections, and cellular bridges [19]. Recently, our laboratory developed a simple, inexpensive method for MCF-7 cell spheroid (solid tumor) growth in 96-well low attachment plates (Figure 1). We used this method to test breast cancer drugs for their effect on the MCF-7 spheroid (Figures 2–6). Our results demonstrate that MCF-7 cell can grow in a plate like a solid tumor, and this method can be used for cancer drug testing and mechanistic studies.

3D cell culture has become more and more a focus of research in cancer drug discovery and development recently [23] [24]. 3D spheroid culture methods recreate more physiologically relevant microenvironments for cells. Therefore, these models are more appropriate for cancer and other disease drug screening. Leveraging recent advances in 3D spheroid technology for disease research and drug development, we developed MCF-7 cell spheroid culture method for the development and discovery of breast cancer drugs. We expect the results of these studies will support the subsequent development of drugs that can prevent tumorigenesis and/or halt the growth of estrogen-dependent breast cancers. This method can also be applied clinically to develop personalized drugs for individual breast cancer patients. This spheroid approach, targeting estrogen metabolizing enzymes and estrogen receptors for the development of estrogen-dependent breast cancer drugs may also lead to the discovery of novel therapy.

**Scheme 1.**
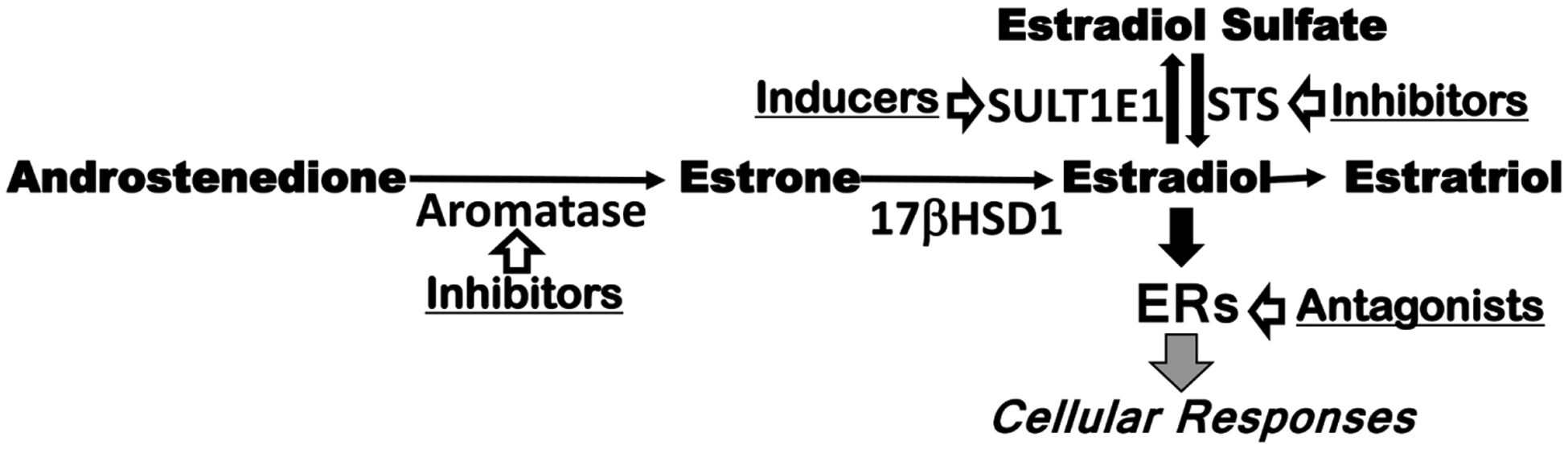
Estrogen pathways and estrogen blocking drugs. SULT1E1 = estrogen sulfotransferase, STS = steroid sulfatase, ER = estrogen receptor.

In females, estrogens are mainly synthesized in the ovaries where they are sulfated by SULT1E1 to increase solubility for release into the circulation. The inactive estrogen sulfates are hydrolyzed by STS to regenerate active estrogens in the targeting tissues (Scheme 1). Estrogens exert their proliferative effects by bind to estrogen receptors. Two nuclear ERs, ER*α* and ER*β*, are relatively well studied for their roles in breast cancers [25] [26] [27] [28] [29]. G Protein-coupled ER (GPER) is a plasma membrane protein, resulting in intracellular calcium mobilization and synthesis of phosphatidylinositol (3,4,5)-trisphosphate. GPER plays roles in the rapid non-genomic signaling events [30] 31] [32] [33]. It is not well studied, and its roles in breast cancer are not known [31] [34] [35]. Our experimental results (Figures 3–6) suggest that GPER may be critical for MCF-7 spheroid growth, while ER*α* and ER*β* may not play vital role in MCF-7 spheroid growth.

Breast cancer is the most commonly occurring cancer in women. It is the leading cause of cancer death among females globally. Majority of breast cancers are estrogen-dependent (60% – 80% based on different publications) [36] [37] [38] [39] [40]. Chemotherapy for breast cancer after surgery (adjuvant chemo) can kill any cancer cells that might have been left behind or have spread but cannot be seen. Adjuvant chemo can lower the risk of breast cancer coming back. Chemotherapy can also be used before surgery (neoadjuvant chemo) to shrink the tumor so that it can be removed with less extensive surgery. Chemotherapy is most effective when combinations of drugs are used [41] [42]. Chemotherapy is an indispensable therapy for the treatment of cancers, especially for estrogen-dependent breast cancers. Certain breast cancer drugs can also be used for the prevention of breast cancers for high-risk family women.

In summary, 3D culture can use patient-derived organoids to develop personalized medicine. It can also use different cancer cell lines to grow tumoroids to develop specific effective drugs for various types of cancers. We developed a new MCF-7 cell spheroid culturing protocol, and used that for drug effect studies. Our current results suggest a very effective and easy method for drug development for cancers. Spheroids have a complex architectural structure, dynamic cell-cell interactions, and very closely mimicking *in vivo* microenvironment. 3D multicellular spheroids have recently emerged as a powerful *in vitro* tool that closely mimic *in vivo* models for new drug development and individualized patient treatment. Our spheroid culture method (see Method section) using regular culture medium, and low-attachment, round bottom culture plates, is inexpensive. The operational protocol is relatively easy (only difficulty is the partial remove of medium by pipetting). Our spheroid culture method will significantly contribute to the cancer new drug development, different types of cancer growth mechanisms, and personalized medicine development. Based on our data, less than one million cells (~1 mg isolated cells) are needed from an individual patient to use this method to develop effective personalized drugs. One thousand cells are enough to develop one spheroid (tumor) in an individual low attachment plate well for drug testing. Our spheroid culture method results suggest that estrogen metabolizing enzyme, SULT1E1 and STS play important roles in MCF-7 cell spheroid growth. G protein-coupled estrogen receptor is critical for MCF-7 cell spheroid growth, while ER*α* and ER*β* may not be critical for MCF-7 cell spheroid growth. These results need to be further proved using molecular biological methods.
